# Hearables: Multimodal physiological in-ear sensing

**DOI:** 10.1038/s41598-017-06925-2

**Published:** 2017-07-31

**Authors:** Valentin Goverdovsky, Wilhelm von Rosenberg, Takashi Nakamura, David Looney, David J. Sharp, Christos Papavassiliou, Mary J. Morrell, Danilo P. Mandic

**Affiliations:** 1 Department of Electrical and Electronic Engineering, Imperial College London, London, SW7 2BT United Kingdom; 2 Computational, Cognitive, and Clinical Neuroimaging Laboratory, Centre for Neuroscience, Division of Brain Sciences, Imperial College London, London, W12 0NN United Kingdom; 3 Academic Unit of Sleep and Ventilation, National Heart and Lung Institute, Imperial College London, London, United Kingdom; 4 NIHR Respiratory Disease Biomedical Research Unit at the Royal Brompton and Harefield NHS Foundation Trust and Imperial College London, London, SW3 6NP United Kingdom

## Abstract

Future health systems require the means to assess and track the neural and physiological function of a user over long periods of time, and in the community. Human body responses are manifested through multiple, interacting modalities – the mechanical, electrical and chemical; yet, current physiological monitors (e.g. actigraphy, heart rate) largely lack in cross-modal ability, are inconvenient and/or stigmatizing. We address these challenges through an *inconspicuous* earpiece, which benefits from the relatively stable position of the ear canal with respect to vital organs. Equipped with miniature multimodal sensors, it robustly measures the brain, cardiac and respiratory functions. Comprehensive experiments validate each modality within the proposed earpiece, while its potential in wearable health monitoring is illustrated through case studies spanning these three functions. We further demonstrate how combining data from multiple sensors within such an integrated wearable device improves both the accuracy of measurements and the ability to deal with artifacts in real-world scenarios.

## Introduction

Recent advances in wearable technology and the Internet-of-Things have opened the possibility for monitoring human physiological functions out of the clinic and in the community. Once fully developed, this is envisaged to significantly alter the landscape of healthcare through continuous 24/7 management of diagnosis and treatment. Early attempts in this direction employed actigraphy, which uses inertial sensors to measure activity of various body parts (BodyMedia), with applications in sports science and general health. More recent efforts have focused on recording cardiac activity, either electrically, via electrocardiograms (ECG), or optically, through photoplethysmograms (PPG). These require either a chest-strap or wrist-band, with the latter considered inconspicuous and comfortable to wear for most people, and the former quite cumbersome.

The development of wearable devices for other modalities, such as respiration and neural activity, has been largely hampered by the inconvenience of their respective form factors – a chest-strap within respiration monitors is uncomfortable for long-term use, while the electroencephalogram (EEG) recorded from the scalp is cumbersome to set up, stigmatizing when out-of-the-clinic, and prone to artifacts. More recent technologies, such as AcuPebble, make use of very sensitive miniature microphones integrated within a wireless device placed over the suprasternal notch, allowing the detection of minute sounds produced by the turbulent airflow in the lungs, as well as heart beats. Such devices are envisaged to find application in the monitoring of sleep apnea, chronic obstructive pulmonary disease, and asthma.

The de facto standard for measuring brain electrical activity is the electroencephalography which involves the placement of a *multielectrode array* onto the scalp and requires an electrolyte to enhance the contact between the electrodes and the skin. Recent efforts to improve this technology have focused on reducing the number of electrodes, exploring new materials (composites^1, 2^, nanowires^3^) and their alternative physical forms (spikes^4^, needles^5, 6^, bristles^7^, capacitive disks^8^), together with producing visually appealing designs (Emotiv). Despite continuous improvements in the areas of electrode design and user-friendliness, the widespread out-of-the-clinic use of head-mounted EEG-devices is unlikely, due to their obtrusive, stigmatizing, and non-discreet nature.

One novel solution for continuous and unobtrusive EEG monitoring uses a curved electrode system integrated on the auricle, with the electrodes operational over two weeks, provided the spray-on bandage is regularly reapplied to the device^9^. The authors state that the electrode placement procedure – critical to ensure high EEG quality – is non-trivial. Furthermore, new form factors – in-ear^10, 11^ and around-the-ear^12^ – have recently emerged which allow the electrodes to be concealed in an inconspicuous EEG acquisition device.

Despite recent advances, the state-of-the-art health monitoring solutions rarely consider more than a single sensing modality. Yet, future health systems require that the whole spectrum of physiological responses is obtained with *as few individual devices as possible*, in order to both provide user convenience and gain additional information through cross-modal coupling. We here introduce such a platform, with the form factor of a standard in-ear headphone, routinely worn worldwide, which integrates miniature sensors for continuously monitoring not only neural, but also cardiac and respiratory activity from inside the ear canal. Together with the key insights from our ealier work on the in-ear sensing of neural activity^10, 13, 14^, the functionality of such a *fully integrated* multimodal sensing platform is demonstrated in multiple real-world scenarios. The proposed device is mechanically stable, unobtrusive, discreet, and straightforward to apply, and, with the advancement of system-on-chip technology, promises widespread continuous physiological monitoring in the community, leading to improved health management.

## Results and Discussion

### Sensor construction

**Figure 1:**
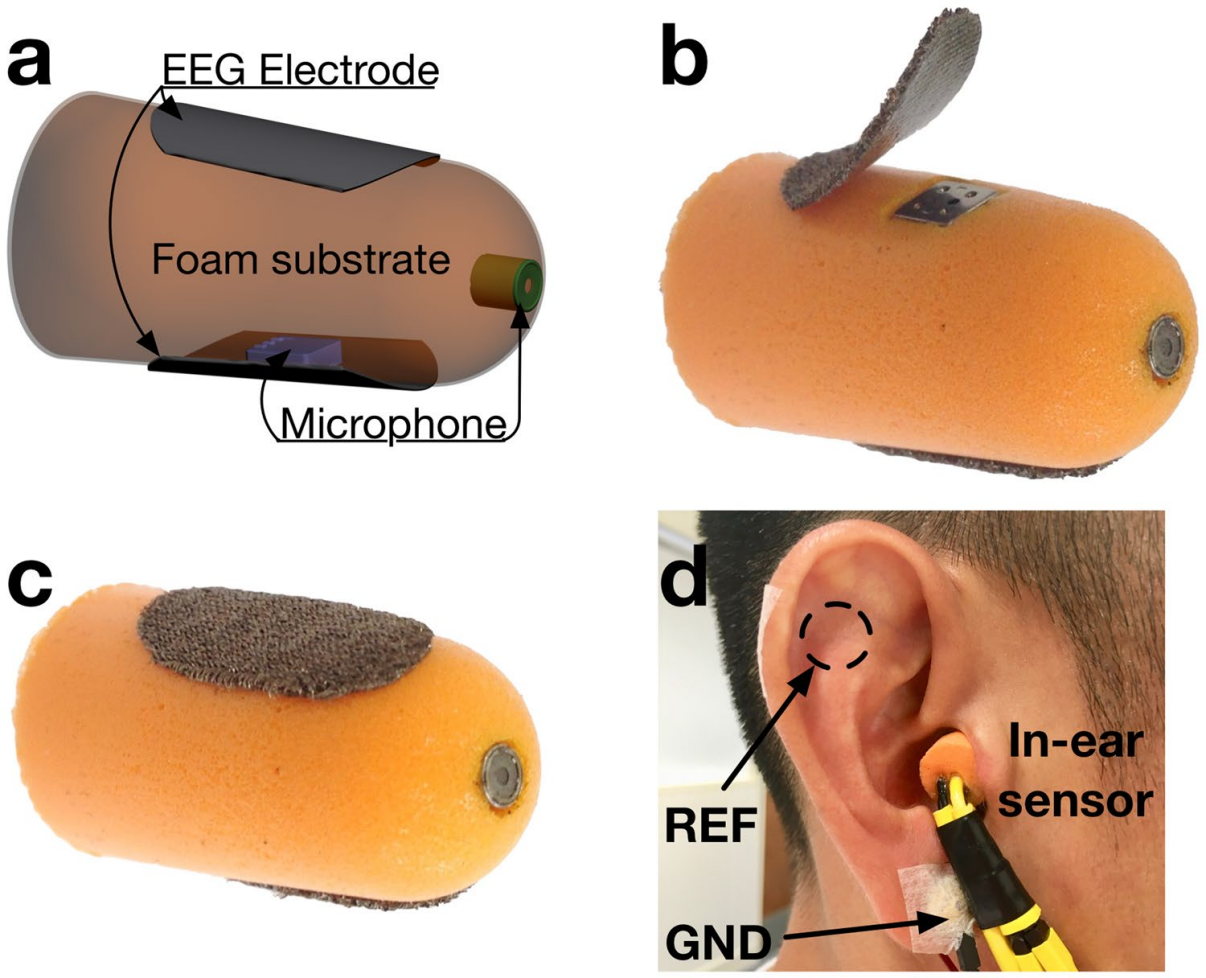
Construction of the multimodal in-ear sensing device. (**a**) Detailed structure of the device, showing the placement of the microphone and the electrode on the substrate. (**b**) Construction of the multimodal sensor underneath one of the cloth electrodes. (**c**) Completed earpiece with electrodes and inward-facing microphone visible. (**d**) Placement of the earpiece in the user’s ear.

**Figure 2:**
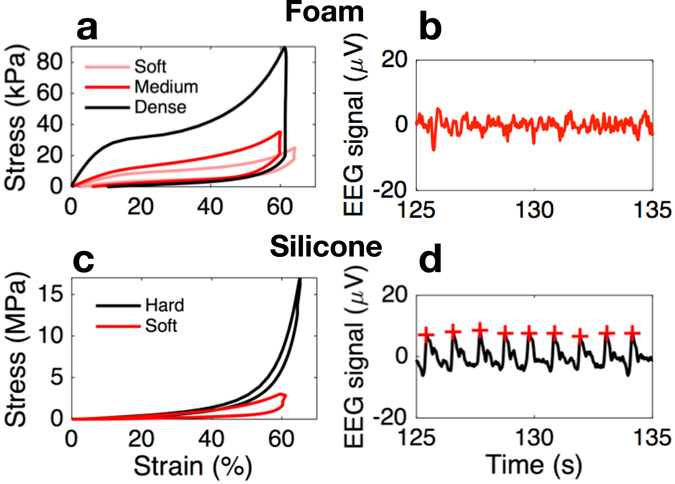
Mechanical tests comparing the viscoelastic foam substrate and silicone^13^. (**a**) Stress-Strain curve for viscoelastic foam cylinders of varying density. (**b**) EEG signal captured with the foam-based earplug from the ear canal of a person with strong blood vessel pulsation, observe no pulsation in the recorded EEG. (**c**) Stress-Strain curve for silicone cylinders of varying hardness. (**d**) EEG signal captured using the silicone-based earplug from the ear canal of the same person with strong blood vessel pulsation, observe strong contamination of the EEG with pulsatile artifacts, indicated by red crosses.

The proposed multimodal generic in-ear sensor comprises five key components: memory-foam substrate, two miniature microphones and two conductive cloth electrodes; Fig. 1a shows details of the device construction. The substrate material is a viscoelastic foam which allows for the absorption of artifacts stemming from both small and large mechanical deformations to the ear canal walls; these may arise from cardiac activity as well as chewing, swallowing and speaking. Furthermore, this flexible earpiece is generic, in the sense that it can be squeezed and shaped to fit any ear. When placed in the ear canal, memory foam relaxes and evenly redistributes the outward pressure throughout its outer surface, thus ensuring a comfortable and snug fit. Fig. 2a,c compare the viscoelasticity of different memory foams with silicone buds – another popular material choice for earplugs. The strain-stress curves were produced for 16 mm tall cylinders of both materials; these were first rapidly (10 mm in 2.4 s) compressed in a dynamic mechanical analyzer, the strain was then held constant for 1 min and finally gradually released at 2 mm/min rate. The two key points to observe are: 1) a dramatic relaxation of the dense foam when held for 1 min at 60% compression (from >80 kPa to <20 kPa), and 2) almost an order of magnitude lower compressive stress in memory foam compared to silicone (kPa vs MPa). These two desirable characteristics ensure comfortable fit and straightforward insertion of the earpieces based on memory foam, together with reduced mechanical artifacts from pulsatile ear canal movements due to blood vessel pulsation, as shown in Fig. 2b,d. Further details of the construction of such a viscoelastic earpiece, as well as its detailed mechanical and electrical characteristics in relation to brain activity monitoring, can be found in ref. 13.

In our proposed device, the electret condenser microphones (ECMs) are integrated within the viscoelastic earpiece to provide two sensing modalities: 1) mechanical disturbance measurements within the multimodal sensor (MMS) in order to deal with motion artifacts, and 2) the ability to record speech and breathing activity from inside the ear canal. The MMS is constructed by first embedding an ECM within the body of the memory foam substrate such that it is aligned with the surface of the earplug, as shown in Fig. 1b. The microphone is subsequently sealed with a thin layer of plastic film, thus providing electrical insulation and mechanical “permeability“. Finally, a cloth electrode is placed over the microphone and attached to the substrate with an adhesive, thus forming a flexible electro-mechanical electrode^13, 15^ capable of co-located sensing of both the desired signal and disturbance; the contralateral electrode is attached to the earpiece in the same way but with no ECM underneath.

Flexible cloth EEG electrodes are constructed out of conductive stretchable knitted fabric with less than $2{\rm{\Omega }}/\square$ of surface resistivity. Fabric is cut into strips of 4 by 7 mm and all sensors on the multimodal earpiece are connected to signal acquisition equipment.

### Physics behind ear-EEG

**Figure 3:**
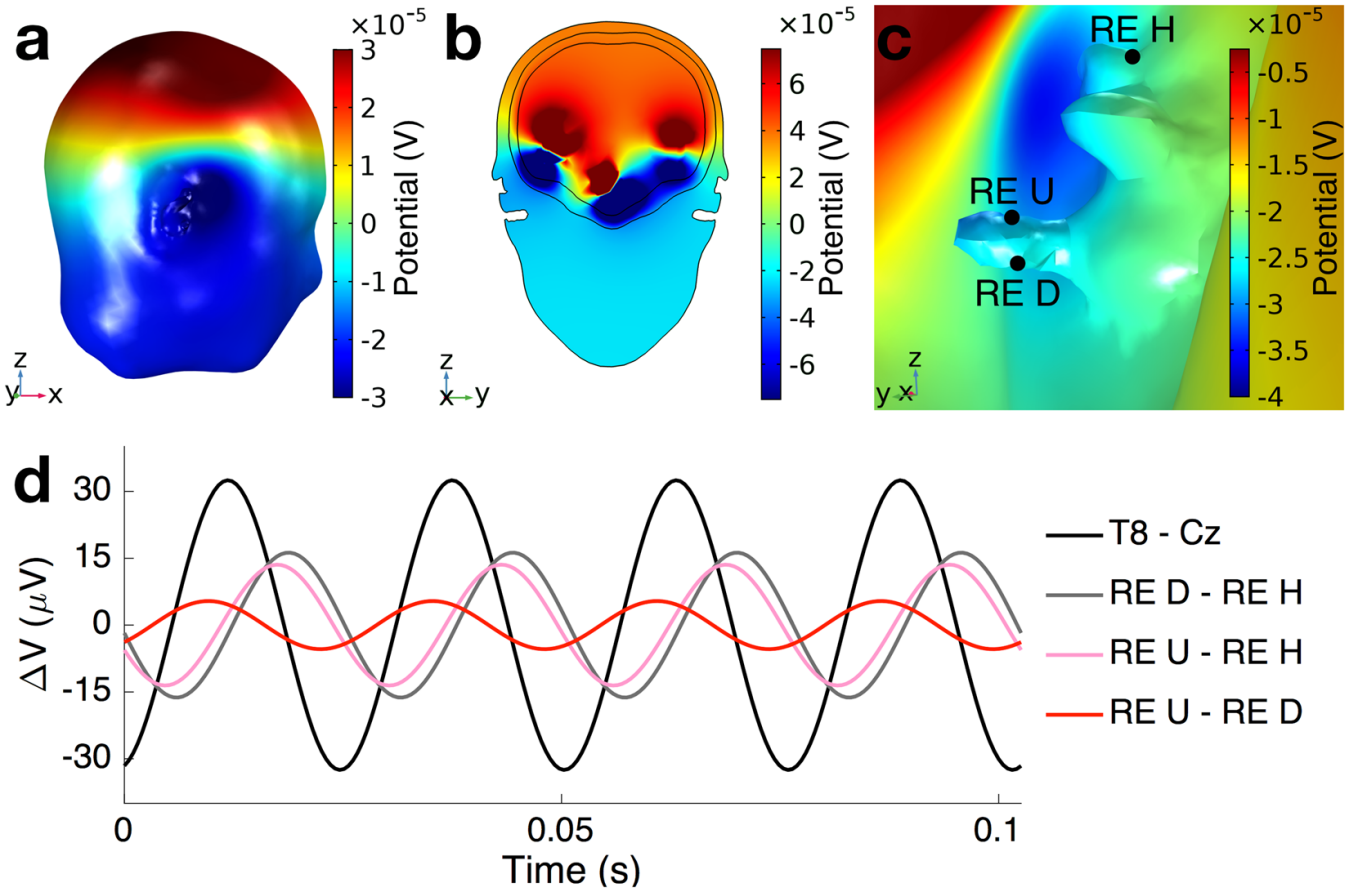
Simulated electric potentials in the head at time 0 s, generated by dipoles which oscillated at 39 Hz, with positive potentials shown in red and negative potentials in blue. (**a**) Potentials on the whole scalp; (**b**) Potentials in the coronal plane; (**c**) Potentials in the right ear region seen from inside the head (note that (**a**–**c**) have different scales); (**d**) Waveforms of potential differences between electrode pairs on the scalp and inside the ear canal. The T8 and Cz positions are standard on-scalp EEG electrodes and further abbreviations are: RE D: Right ear canal, downward direction; RE U: Right ear canal, upward direction; RE H: Right ear, root of the helix. The positions of the ear electrodes are marked with black circles in panel (**c**).

Unlike the propagation of the electric field from brain sources to the scalp, the propagation from brain sources to the ear canal is not well understood. In order to establish a feasibility model for the measurement of EEG from inside the ear canal, biophysics simulations were conducted in COMSOL Multiphysics software. For rigour, *a realistic three*-*shell head model was generated from MRI scans* to enable a meaningful comparison of scalp- and ear-EEG amplitudes – not possible to achieve with previously used idealised one-shell two-dimensional models^16^. The auditory steady-state response (ASSR) was modeled so as to arise from the superposition of electric fields produced by six current dipoles^17^, two in *each* auditory cortex and two in the brainstem, see Methods. Fig. 3a–c show the estimated brain electric potentials at time 0 s with respect to the phase onsets; the upper part of the head was positively charged (red) while the lower part was charged negatively (blue). Locations of the electrodes were chosen to be inside the ear canals, root of the helix, and at the standard scalp positions Cz (central) and T8 (temporal lobe).

**Figure 4:**
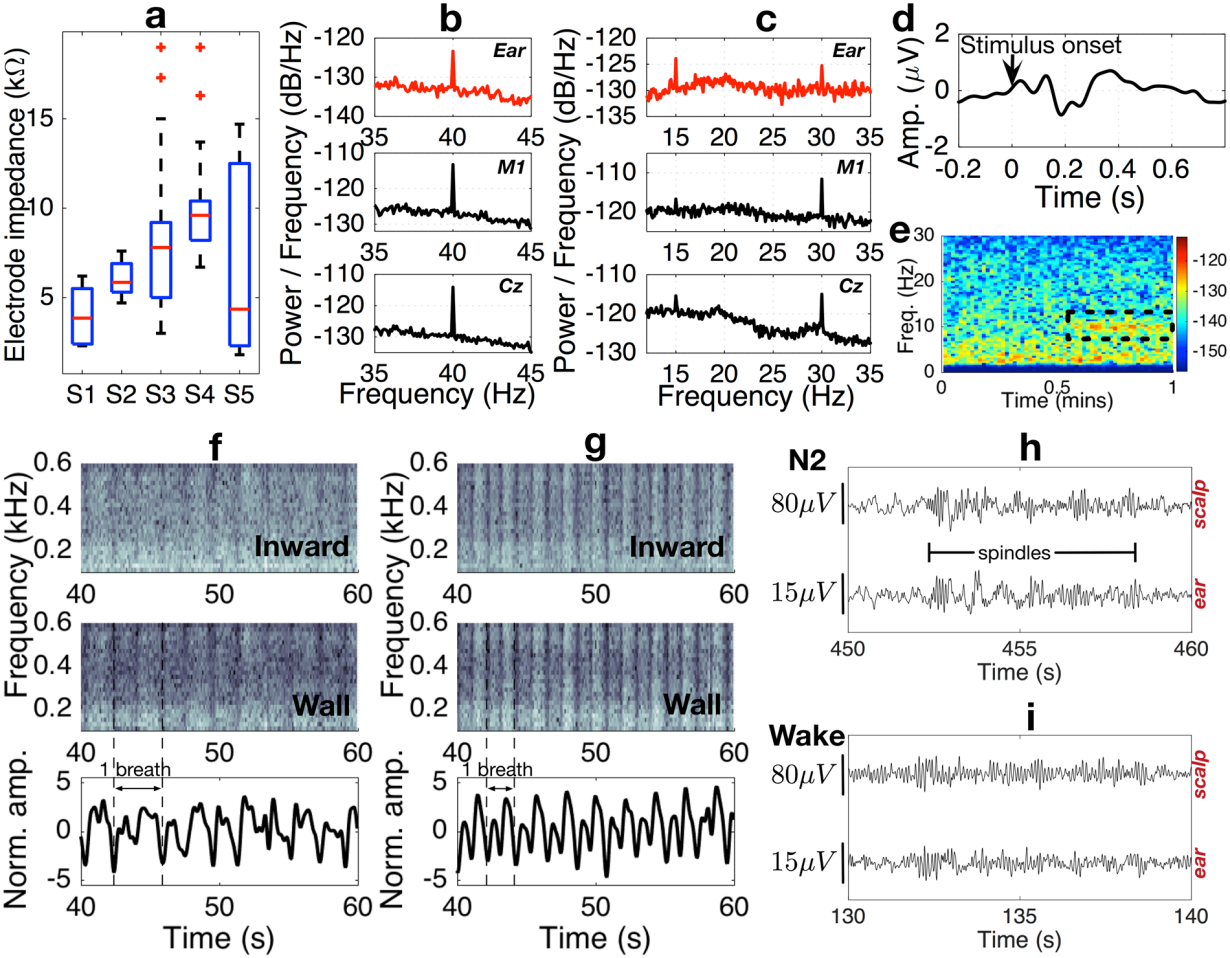
Acquisition of EEG and respiration from inside the ear canal. (**a**) Stability of the electrode-skin interface over the course of 8 hours, for 5 subjects^13^. (**b**) ASSR response measured from the ear, M1 and Cz scalp locations. (**c**) SSVEP response measured from the ear, M1 and Cz locations. (**d**) Visual evoked potential measured with the earpiece^13^. (**e**) Alpha rhythm recorded from the ear electrodes, 30 s into the trial subject closed their eyes. (**f**,**g**) (Top and Middle) Spectrograms of acoustic signals measured by the two microphones (inward- and wall-facing) integrated within the earpiece when the subject was asked to breath at 16 and 28 breaths per minute, respectively. (**f**,**g**) (Bottom) Breathing signals extracted from the associated spectrograms. (**h**) EEG signals from scalp and ear canal during the N2 stage of sleep. (**i**) EEG signals from scalp and ear canal when the subject was awake, but with eyes closed.

Fig. 3d shows the resulting EEG amplitudes from the scalp and ear canal electrodes; four full cycles of the simulated EEG are shown. While the exact potential differences for a given electrode pair depend on individual head geometries and the orientation and strength of EEG-generating dipoles, the amplitude is largest for the T8 – Cz scalp electrode pair. In comparison to the T8 – Cz amplitude, the amplitudes from electrode pairings between the root of the helix and either of the in-ear positions are approximately half as large, and from the two in-ear electrodes approximately a sixth. Due to shorter electrode distances and the ASSR set-up, the ear-EEG amplitudes in Fig. 3d were expected to be weaker than those recorded from the scalp, as experimentally confirmed in Fig. 4b. This clearly demonstrates the feasibility of the EEG acquisition from inside the ear canal.

### EEG acquisition from the ear canal

Our multimodal earpiece was designed to be a comfortable and unobtrusive EEG monitor^13^. To ensure high signal fidelity, its electrodes must provide low contact impedance with the skin over a prolonged period of time. The choice of electrode material – low impedance stretchable fabric – ensures that this requirement is satisfied even when only small amount of saline solution is applied to the electrodes prior to insertion; the placement of the device inside the ear canal also ensures the desired low evaporation rates. Fig. 4a illustrates the stable nature of the electrode-skin impedance measured at 30 min intervals for 5 subjects over the course of their normal 8-hour working day in the office, with no restrictions placed on their activities which included having lunch and talking to people. Observe that, as desired, in all cases the median electrode impedance remained quite low, below 10 kΩ.

Having demonstrated good and stable electrode impedances, we proceeded to test the device over a number of well established EEG paradigms: ASSR, steady-state visual evoked potential (SSVEP), transient response to visual stimulus (VEP), and alpha rhythm, as summarized in Fig. 4b–e. The ASSR at a 40 Hz modulation frequency was obtained from both ear-EEG, the mastoid (M1), and the central scalp (Cz) electrodes, and compared in Fig. 4b. Observe clear peaks at the frequency of 40 Hz for all the recording positions – the signal to noise ratio (SNR) of the EEG from the ear canal was similar to that from conventional on-scalp electrodes.

The SSVEP was induced in EEG by presenting the subjects with an LED blinking at 15 Hz. As desired, a clear peak was observed at the stimulus frequency of 15 Hz and also at its first harmonic at 30 Hz. Since the response was distributed across multiple harmonics, it was not straightforward to quantify the SNR of the recorded SSVEP, however, qualitatively it is evident that the response from the ear electrode was weaker than that of scalp electrode from a central brain region. This behaviour was expected due to a larger distance between the EEG source in the occipital region and the ear canal, as well as because of smaller electrode distances within ear-EEG, and supports the biophysics propagation model in Fig. 3.

We further demonstrated the functionality of the proposed device to acquire transient neural responses by presenting subjects with an LED switched fully ON for 200 ms and then fully OFF for 1800 ms. This response manifests itself in the negative deflection of the EEG signal approximately 180 ms after stimulus onset when recorded from the mastoid electrodes. Fig. 4d demonstrates that the shape and timing of the VEP waveform, as measured from the ear electrode, are a good match for the corresponding waveforms from scalp electrodes^18^.

Another aspect of the proposed multimodal earpiece is the ability to predict and assess the fatigue based on the neural activity in the alpha band^19, 20^, which is usually associated with the state of wakeful relaxation and manifests itself in the EEG oscillations in the 8–12 Hz frequency range, centered around 10 Hz. The loss of alpha rhythm is also one of the key features used by clinicians to define the onset of sleep. To demonstrate the capability of our device in measuring the alpha rhythm, the subjects were asked to sit still with the eyes open and then to close their eyes approximately 30 s into the recording. The time-frequency plot of the resulting EEG for one of the subjects is shown on Fig. 4e. The increase in the power of the alpha band is apparent after the subjects closed their eyes, evidenced in the black dashed box. Additional details and discussion of the neural activity monitoring with the generic viscoelastic earpiece can be found in ref. 13.

### Dealing with EEG artifacts through multimodality

**Figure 5:**
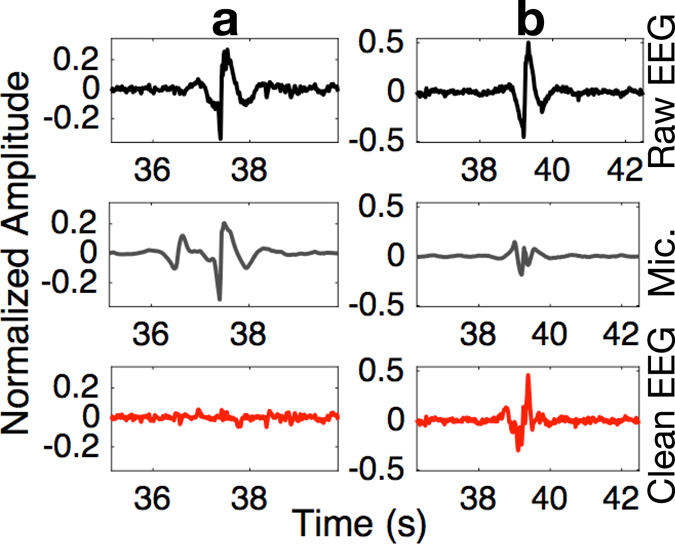
Denoising of ear-EEG from mechanical jaw clench artifacts. (**a**) Best-case denoising scenario for which the artifact measurement with the embedded microphone was accurate. (**b**) Worst-case accuracy of artifact measurement with the embedded microphone, note that the artifact was nevertheless reduced in the *Clean EEG* trace. (Top panels) Raw EEG corrupted by a strong artifact. (Middle panels) Output of the mechanical sensor within the MMS. (Bottom panels) Denoised EEG using the mechanical MMS signal as reference.

We have demonstrated in Fig. 2 that the viscoelastic earpiece, by the very nature of its substrate, helps mitigate the component of electrical noise in EEG stemming from mechanical movement of the ear canal walls against the electrode, the so called *motion artifact*. However, such noise cannot be completely suppressed by the properties of the substrate alone, particularly during strong jaw clenches, as shown in Fig. 5 (Top panel). To deal with a variety of stronger artifacts, as typically occur in real-world scenarios, the proposed earpiece integrates a mechanical transducer (electret condenser microphone) within its multimodal electro-mechanical sensor, which can be used as a reference for single-channel digital denoising of physiological signals^21, 22^. The results of such denoising in the jaw clenching scenario – when the microphone signal corresponding to the artifact is very accurate – are illustrated in Fig. 5a. Observe a significant reduction in the level of jaw clenching artifacts in the ear electrodes. In another, less favorable scenario (Fig. 5b), the proxy of the artifact measured by the ECM was compromised, but nevertheless mechanical noise was considerably attenuated. This is the first time that the possibility of removing *real*-*world motion artifacts* from ear-EEG, with the aid of a multimodal sensor embedded within the earpiece, has been verified and shown to work in a real-life scenario, and is a major step forward in our endeavor to measure neural activity outside the lab with no constraints imposed on the actions of the user.

### Speech

The miniature bone conduction microphone within the proposed multimodal earpiece is specifically designed for measuring acoustic signals traveling through dense tissues of the head. The original ECM transducer was modified by covering its acoustic hole with a layer of insulating material and a layer of conductive material, both of which are flexible, so that the ECM retains ability to capture acoustic signals. To enhance the capabilities of the multimodal earpiece in capturing speech and breathing signals, we have also placed an even smaller ECM at the tip of the earpiece facing towards the eardrum. This microphone directly captures acoustic energy traveling from the vocal chords via auditory tube to the ear canal. The output of such a microphone would be expected to provide better speech quality than the sealed microphone within the multimodal sensor.

**Figure 6:**
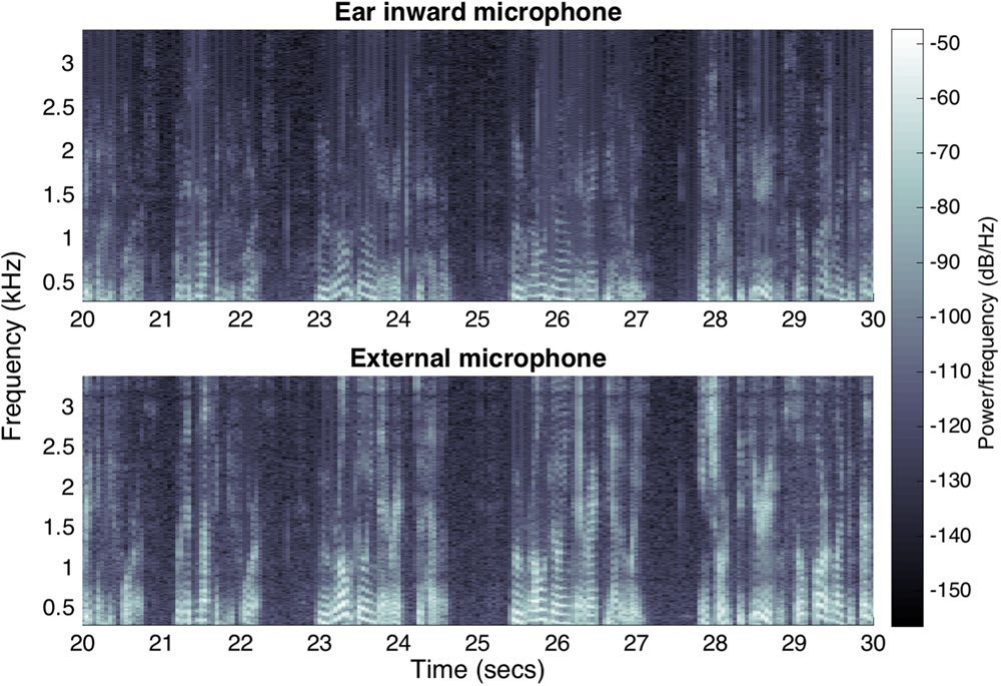
Recording of speech from the multimodal earpiece, illustrated through a 10 s spectrogram. (Top) Speech signal recorded using a microphone placed on the tip of the earpiece looking towards the eardrum. (Bottom) Speech signal from an external microphone placed on a subject’s chest. The Y-axis for both spectrograms spans the 300–3400 Hz band, the so-called voice frequency band.

In the experiment subjects were asked to read out loud in their normal voice and tempo the first paragraph from the Charles Dickens’ *A Tale of Two Cities*, and the resulting audio signals were simultaneously measured using: 1) an external microphone placed on the chest, 2) the multimodal sensor within the earpiece, and 3) the inward facing microphone integrated within the proposed earpiece, as shown in Fig. 1. The spectrograms of 10 s duration are given in Fig. 6 and the resulting speech recording for one of the subjects is available in the electronic supplementary material.

A standard technique for assessing the speech quality is cepstral distance^23^ (CD), which for the simultaneous recordings by two identical external microphones was found to be 0.56, the figure which corresponds to the ideal case of two almost identical speech waveforms. The CD between an external microphone and the embedded microphone placed inside the ear canal looking towards the eardrum, as well as the microphone within the MMS, was found to be 0.90, indicating that quality of the speech picked up from the earpiece inside the ear canal was similar to that obtained with the microphone placed externally. To put this finding into a broader context, the CD between white Gaussian noise and an external microphone was approximately 2.11. A somewhat larger CD of speech from the in-ear microphones versus external microphone can be explained by the dampening of the high-frequencies of the speech signal caused by its propagation through the skull and other tissues of the head, which did not affect the intelligibility.

### Respiration

In addition to speech, the proposed multimodal earpiece is capable of measuring another, more challenging, acoustic signal present inside the ear canal – the respiration. Breathing creates turbulence within the airways, so that the turbulent airflow can be measured using a microphone placed externally on the upper chest at the suprasternal notch^24^. Albeit weak, such sounds also travel through the tissues of the head and the auditory tube^25^. To prove that respiration signatures can be measured not only with the inward-facing microphone, but also with the ECM within the multimodal sensor four subjects were asked to breath in time with a metronome at different rates. At low rates (4 and 8 breaths per minute (brpm)) the acoustic signal in the ear was too low to be measured reliably by the ECMs on the earpiece. As the respiration rate increased, more turbulence was produced which increased the power of the breathing-related sound within the ear; starting from breathing rates of approximately 12 brpm we could reliably measure the breathing rates, as shown in Fig. 4f (16 brpm) and 4g (28 brpm).

The respiratory signals recorded inside the ear canal are weak, and are thus expected to be affected by motion artifacts arising from a significant movement of the earpiece inside the ear canal. We envisage that this issue can be mitigated through a control loop which involves knowledge of the degree of artifacts and total output power from the microphones.

### Cardiac activity

**Figure 7:**
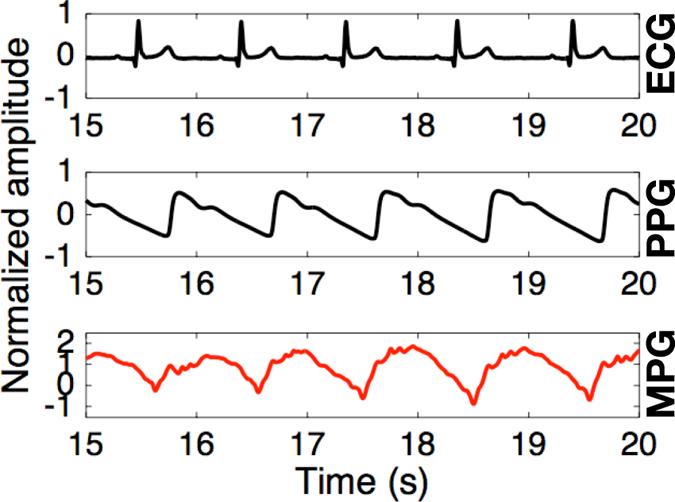
Cardiac activity captured from inside the ear canal using the multimodal sensor embedded within the earpiece. (Top) ECG signal from the arms. (Middle) PPG signal from the finger. (Bottom) Integral of the mechanical signal produced by the microphone within the multimodal sensor.

Another task of the multimodal sensor integrated within the earpiece was to sense minute mechanical movements of the tissues under the skin of the ear canal which result from blood vessel pulsation. To verify this capability, we set up an experiment which compared the cardiac activity recorded from the proposed earpiece with the benchmark ECG obtained from the hands and the standard PPG from a finger. Fig. 7 illustrates the recorded waveforms; observe that the integral of the pulsatile waveform from the multimodal sensor (mechanical plethysmography (MPG)) resembles the waveform from the PPG sensor. The accuracy of the measured heart rate was similar across all three sensors, as exemplified by the respective ECG-PPG and ECG-MPG Pearson correlation coefficients of 0.98 and 0.99. Overall, this verifies that, in the absence of jaw movements, the proposed earpiece can robustly measure the pulse rate and provide a proxy to the PPG waveform, in a more convenient way than is current practice.

Although it has been reported that a PPG sensor can be integrated on an earpiece^26^, our multimodal sensor offers several advantages. Firstly, it has very low power consumption: typical current draw of the ECM is only 35 *μ*A. Secondly, unlike the PPG sensor, it can directly measure acoustic signals in the ear canal. Thirdly, as shown in Fig. 5, its output can be used to denoise the EEG corrupted by motion artifacts. All in all, the proposed device combines several important capabilities in a miniature package, and without the need for additional components, which is an important consideration due to extremely constrained space available inside the ear canal.

### Scoring sleep from ear-EEG

We have shown so far that the proposed device is capable of measuring a variety of EEG responses – alpha rhythm, ASSR, SSVEP and VEP – as well as multiple mechanical signals associated with cardiac activity, speech and breathing. One of the key areas where such a device may find application is in monitoring sleep, assessing its quality, and possibly sleepiness. Currently, this is performed using cumbersome sleep electrodes^27^.

For a proof of concept of the utility of our device in sleep scoring based on EEG in particular, we recruited four subjects who were asked to reduce their sleep the night before the experiment to no more than 5 hours. On the day of the experiment the subjects slept in the afternoon for 45 minutes, with scalp and ear electrodes simultaneously recording EEG^14^. The scalp- and ear-EEG signals were blinded, appropriately re-scaled, filtered and presented to sleep clinicians for scoring. Example traces from the N2 and Wake stages of sleep are given in Fig. 4h,i for scalp and ear electrodes, while Table 1 shows the scores of 360 epochs of nap EEG.

**Table 1:** Contingency table of epochs for the Wake versus Sleep stages (N1, N2 and N3) for all subjects.

	Wake [ear]	N1 [ear]	N2 [ear]	N3 [ear]
Wake[scalp]	70	19	17	0
N1[scalp]	15	20	19	0
N2[scalp]	7	17	120	9
N3[scalp]	0	1	25	21

The agreement between the sleep scores from the scalp and the ear canal in distinguishing wake (Wake) from sleep (N1, N2 and N3 combined) was quantified through the kappa coefficient; its value of 0.60 (95% confidence interval 0.5 to 0.69) indicates a substantial agreement. This illustrates the promise of the proposed device in out-of-hospital assessment and diagnosis of sleep-related conditions, such as insomnia, sleep apnea, and excessive daytime sleepiness, please see ref. 14 for more information and in-depth analysis of the utility of ear-EEG in sleep monitoring.

Wearable technology is envisaged to revolutionize the way we approach health and well-being, but for this to become a reality, multiple physiological sensors should be integrated into a single, comfortable-to-wear, unobtrusive and non-stigmatizing package. By integrating a number of miniature sensors on an earpiece, we have demonstrated the wide-ranging potential of multimodal in-ear sensing in measuring not only neural, but also cardiac, speech and respiratory activity in an inconspicuous and comfortable manner. The viscoelasticity of the substrate helps mitigate the adverse effects of mechanical disturbances (motion, blood vessel pulsation) on signal quality, a critical issue in out-of-clinic EEG. Through the cross-modal information, the device has been shown to deal with even the most challenging real-world artifacts – jaw-clenches. The potential of the earpiece has been validated through case studies ranging from event related potentials, cardiac and respiratory activity, through to real-world application of sleep monitoring. Future work will consider integration within the earpiece of the means for signal acquisition, preprocessing, and wireless communication.

## Methods

### Physics simulations

The work in ref. 17 examined the mechanisms of eliciting ASSR in EEG for three amplitude-modulating frequencies, 12 Hz, 39 Hz, and 88 Hz, and localized the ASSR as arising from three different sources, with two dipoles of different orientations and amplitudes per source (six components in total). The so–identified sources of ASSR were located in: (i) the brain stem, comprising a vertical and a lateral (from the left to the right) source component, (ii) the left auditory cortex, and (iii) the right auditory cortex, with both (ii) and (iii) having a tangential and a radial component. The current dipole sources employed in our model, together with their relative amplitudes and phases with respect to the stimulus onset, were placed accordingly. For rigor, a three-shell head model with realistically shaped geometries was created, whereby the shell geometries were obtained by segmenting a real-world magnetic resonance image (MRI). The geometry of the inner ear was based on a 3D scan of an earmold. In the simulations, the biophysical model of the head was surrounded by a sphere of radius 1 m filled with air, while the tissue properties were taken from the IFAC-CNR^28^ based on data published in refs 29–31. The COMSOL Multiphysics 5.2 software was used for the biophysical simulations.

### Physiological measurement experiments

In all the experiments, signal acquisition was performed using the g.tec g.USBamp amplifier with a 24-bit resolution and a sampling rate of 1200 S/s. The ASSR stimulus comprised a 1 kHz tone, amplitude-modulated with a 40 Hz sinusoidal signal at a 100% modulation level. The numbers of volunteers involved in different measurement experiments were: SSVEP and VEP – 3, Alpha rhythm – 4, EEG denoising – 6, Speech and breathing – 4, Nap recordings – 4, Cardiac activity – 3. Sleep experiments used standard scalp electrode positions C3 and C4 referenced to A2 and A1 respectively, the ground electrode was placed on the forehead. All the experiments involving ear-EEG had the two ear electrodes referenced to a gold cup electrode placed behind the helix of the same ear, while the ground was placed on the earlobe of the same ear. All the experiments were performed at Imperial College London, under ethically approved protocol, reference ICREC 12_1_1, Joint Research Office at Imperial College London. Informed consent was obtained from all subjects and all experiments were performed in accordance with the approved guidelines and regulations.

### Preprocessing of signals

The ASSR and SSVEP response signals were filtered to the 1–45 Hz range using a 4th order Butterworth band-pass filter, with zero-phase filtering. The same filter was utilized for pre-processing alpha rhythm, VEP, and sleep recordings, but with 1–20 Hz, 1–13 Hz and 1–20 Hz bandwidths respectively. Breathing signals were filtered with a 10th order Butterworth filter to the frequency range of 100–600 Hz, while the corresponding spectrograms used a sliding Hamming window of 0.2 s with 50% overlap.

### Speech signal acquisition and processing

Speech signals were acquired using the three available microphone inputs on Apple Mac mini (late 2014 model). The sampling rate was set to 40 kHz. For calculating the spectrogram and the cepstral distance, signals were downsampled to 20 kHz. Cepstral distance was calculated following the procedure outlined in ref. 23, with segment lengths of 512 samples and based on the following definition for the cepstrum: ${|IDFT(log({|DFT(x)|}^{2}))|}^{2}$.

### Personally identifiable information

Informed consent to publish in an open-access publication, has been obtained from the individual, whose ear appears in the photo in Fig. 1. This manuscript contains no other personally identifiable information.

## Electronic supplementary material

Supplementary video 1

Supplementary video 2

Supplementary video 3
